# Supplementary material for: Identification of photoperiod-induced specific miRNAs in the adrenal glands of Sunite sheep (Ovis aries)
Source: Front Vet Sci. 2022 Jul 22;9:888207. doi: 10.3389/fvets.2022.888207 (PMC9354845; doi:10.3389/fvets.2022.888207)
Supplement: Supplementary file 1 [file Data_Sheet_1.ZIP › Supplementary file/miRDeep2 file.zip!/miRDeep2 file/Know_Structure/SPLP42A3/oar-mir-655.pdf]

miRBase precursor : oar-mir-655
Total read count : 1194
oar-miR-655-5p read count : 4
oar-miR-655-3p read count : 1190
remaining reads : 0

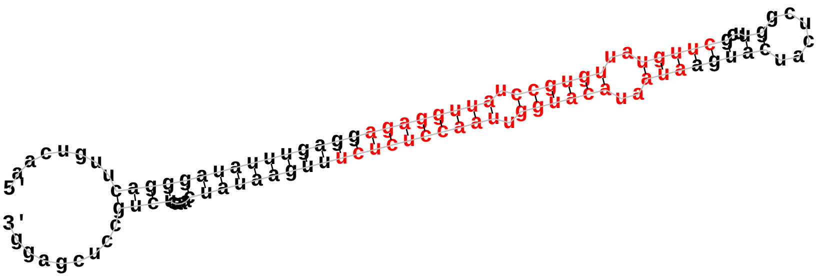

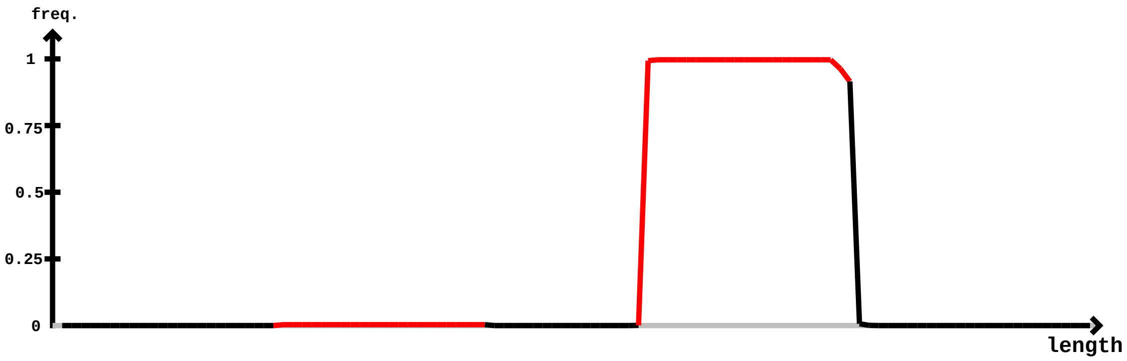


```
                                oar-miR-655-5p
                                                             oar-miR-655-3p
5'- aacuguucagggauauuugaggagagguuauccguguuauguucgcuuggcucaucaugaauaauacaugguuaaccucucuuugaauaucaaacucugccucgagg   -3'    exp
    ...........(((((((((((((((((((.((((((..((((((..((......))))))))..)))))).)))))))))))))))))))...(((......))).     reads     mm      sample
    ......................agagguuauccguguuauguuc...............................................................     4         0       seq
    ...........................................................aauaauacaugguuaaccucucu.........................     1         0       seq
    ............................................................auaauacaugguuaaccucu...........................     39        0       seq
    ............................................................auaauacaugguuaaccucuc..........................     57        0       seq
    ............................................................auaauacaugguuaaccucucu.........................     1081      0       seq
    ............................................................auaauacaugguuaaccucucuu........................     7         0       seq
    ............................................................auaauacaugguuaaccucucuuu.......................     1         0       seq
    .............................................................uaauacaugguuaaccucu...........................     1         0       seq
    .............................................................uaauacaugguuaaccucucu.........................     3         0       seq
```
